# Supplementary material for: An image-based data-driven analysis of cellular architecture in a developing tissue
Source: eLife. 2020 Jun 5;9:e55913. doi: 10.7554/eLife.55913 (PMC7274788; doi:10.7554/eLife.55913)
Supplement: Supplementary file 1. — The number of primordia (N) and of segmented single cells (n) that was used in the analysis for each combination of fluorescent labels. The numbers shown do not include the eight samples that were discarded due to low segmentation quality. [file elife-55913-supp1.docx]

### Supplementary File 1: Table of Dataset Composition

| **Genotype** | **Structure** | **N Primordia** | **n Cells** |
| --- | --- | --- | --- |
| *cldnb:lyn-EGFP* | membranes | 24 | 2310 |
| *cldnb:lyn-EGFP*  *cxcr4b:NLS-tdTomato* | membranes  nuclei | 20 | 2528 |
| *cldnb:lyn-EGFP*  *Actb2:mKate2-Rab11a* | membranes  recycling endosomes | 19 | 1554 |
| *cldnb:lyn-EGFP*  RNA: *mKate2-Rab5a* | membranes  early endosomes | 14 | 1131 |
| *cldnb:lyn-EGFP*  RNA: *mKate2-GM130(rat)* | membranes  *cis*-Golgi | 11 | 866 |
| *cldnb:lyn-EGFP*  *LexOP:CDMPR-tagRFPt*  *cxcr4b:LexPR (driver)* | membranes  TGN & late endosomes | 13 | 967 |
| *cldnb:lyn-EGFP*  *LexOP:B4GalT1(1-55Q)-tagRFPt*  *cxcr4b:LexPR (driver)* | membranes  *trans*-Golgi | 10 | 789 |
| *cldnb:lyn-EGFP*  *atoh1a:dtomato* | membranes  *atoh1a* expression | 14 | 1524 |
| *cldnb:lyn-EGFP*  *6xUAS:tagRFPt-UtrCH*  *ETL GA346 (driver)* | membranes  F-actin | 19 | 1876 |
| *cldnb:lyn-EGFP*  LysoTracker^TM^ Deep Red staining | membranes  lysosomes | 21 | 1802 |
| **Total** |  | **165** | **15347** |
| Additional smFISH dataset (fixed): | | | |
| *cldnb:lyn-EGFP*  *pea3* smFISH staining | membranes  *pea3* RNA molecules | 31 | 3149 |
