# Supplementary material for: An image-based data-driven analysis of cellular architecture in a developing tissue
Source: eLife. 2020 Jun 5;9:e55913. doi: 10.7554/eLife.55913 (PMC7274788; doi:10.7554/eLife.55913)
Supplement: Supplementary file 2. — Brief descriptions of engineered features that were extracted from each segmented cell and used for analysis, in particular to assign biological properties to embedded features extracted with ISLA-CBE. [file elife-55913-supp2.docx]

### Supplementary File 2: Table of Engineered Features

| **Name** | **Description** |
| --- | --- |
| Y,X,Z Centroid Position | Position of cell centroids in TFOR. |
| Orientation along X,Y,Z | Normalized components (in TFOR) of the vector pointing from the cell centroid to the most distant point in the cellular point cloud. |
| A/B Axis Eccentricity | Eccentricity ratios of an ellipsoid fitted to the cellular point cloud. Major, medium and minor (A, B) refer to the length of the principal semi-axes, from largest to smallest. |
| A Axis Length | Length of the ellipsoid's principal semi-axes (A) in microns. |
| A/B Aspect Ratio | Ratios of cell extents along different axes (A, B) in TFOR. |
| X,Y,Z Axis Length | Cell extents along different axes in TFOR, in microns. |
| Longest Extension | Distances from the cell centroid to the most distant point in the cellular point cloud, in microns. |
| Volume | Cell volume (in cubic microns). |
| Surface Area | Cell surface area (in square microns) |
| Sphericity | Measure of how closely the distribution of the cellular point cloud approximates a sphere. It is the mean distance of points to a sphere centered on cell centroid with a radius equal to the mean distance of points from the centroid. The measure is linearly normalized to between 0 and 1, where 1 means perfectly spherical and <1 means less spherical. |
| Roundness (Smoothness) | Measure of how closely the distribution of the cellular point cloud approximates a circumscribed ellipsoid and hence how smooth the surface is. It is the mean distance of points to an ellipsoid fitted to the cloud. The measure is linearly normalized to between 0 and 1, where 1 means perfectly round/smooth and <1 means less round/smooth. |
| Control: Random Normal | A randomly sampled normal distribution with *µ=0* and *σ=1*. Used as a simple "negative control" for any code related to correlation measurements. |
